# The Impact of Height during Childhood on the National Prevalence Rates of Overweight

**DOI:** 10.1371/journal.pone.0085769

**Published:** 2014-01-22

**Authors:** Paula van Dommelen, Marlou L. A. de Kroon, Noël Cameron, Yvonne Schönbeck, Stef van Buuren

**Affiliations:** 1 Department of Life Style, TNO, Leiden, The Netherlands; 2 Department of Public and Occupational Health, Institute for Research in Extramural Medicine, VU University Medical Center, Amsterdam, The Netherlands; 3 School of Sport, Exercise and Health Sciences, Loughborough University, Loughborough, United Kingdom; 4 Department of Child Health, TNO, Leiden, The Netherlands; 5 Department of Methodology and Statistics, Utrecht University, Utrecht, The Netherlands; Northeast Ohio Medical University, United States of America

## Abstract

**Background:**

It is known that height and body mass index (BMI) are correlated in childhood. However, its impact on the (trend of) national prevalence rates of overweight and obesity has never been investigated. The aim of our study is to investigate the relation between height and national prevalence rates of overweight and obesity in childhood between 1980, 1997, and 2009, and to calculate which fixed value of *p* (2.0,2.1, …,3.0) in kg/m^p^ during childhood is most accurate in predicting adult overweight.

**Methods and findings:**

Cross-sectional growth data of children from three Dutch nationwide surveys in 1980, 1997, and 2009, and longitudinal data from the Terneuzen Birth Cohort and the Harpenden Growth Study were used. Relative risks (RR) and 95% confidence intervals (CI) were calculated. Our study showed that tall (>1 standard deviation (SD)) girls aged 5.0–13.9 y were more often overweight (RR = 3.5,95%CI:2.8–4.4) and obese (RR = 3.9,95%CI:2.1–7.4) than short girls (<−1 SD). Similar results were found in boys aged 5.0–14.9 y (RR = 4.4,95%CI:3.4–5.7 and RR = 5.3,95%CI:2.6–11.0). No large differences were found in the other age groups and in comparison with children with an average stature. Tall boys aged 2.0–4.9 y had a significantly higher positive trend in overweight between 1980 and 1997 compared to short boys (RR = 4.0,95%CI:1.38–11.9). For other age groups and in girls, no significant trends were found. The optimal Area Under the Curve (AUC) to predict adult overweight was found for *p* = 2.0.

**Conclusions and significance:**

Tall girls aged 5.0–13.9y and tall boys aged 5.0–14.9y have much higher prevalence rates of overweight and obesity than their shorter peers. We suggest taking into account the impact of height when evaluating trends and variations of BMI distributions in childhood, and to use BMI to predict adult overweight.

## Introduction

Overweight and obesity, and their associated risks of morbidity and early mortality, are considered as rapidly growing public health problems [1]–[2]. Monitoring (trends in) prevalence rates of overweight and obesity within and between countries is important to establish priorities in public health policy. Calculation of overweight and obesity should preferably be based on (inter-)nationally comparable measures. A popular and widely accepted international definition for overweight and obesity is BMI with cut-offs of 25 and 30 kg/m^2^ respectively in adults and the International Obesity Task Force (IOTF) cut-offs during childhood [3].

It is known that BMI is positively correlated with height during childhood [4]–[7]. Some authors have offered explanations for this phenomenon. It is hypothesized that biological processes, nutrition and genes cause early increases in both height and BMI in childhood. This is supported by the finding that height in childhood has been positively associated with skinfold thickness, body fat percentage, obesity, impaired glucose tolerance, leptin levels, insulin resistance, and type 2 diabetes in childhood or in adult life [4]–[15].

As an alternative for childhood BMI, one can use the Benn index (kg/m^p^) as an indicator for adiposity, with *p* chosen such that the index is independent of height. The optimal value of *p* is 2 in pre-school children, increases gradually to 3 at age 11 and falls back to 2 after puberty [16]. The Bogalusa heart study showed that this index is not superior to BMI as an indicator of adiposity and related cardiovascular risk factors during childhood [17]. Moreover, in clinical practice this index is more difficult to apply than BMI since the value *p* depends on age and sex.

Notwithstanding the fact that height and BMI are correlated in childhood, its impact on the (trend of) national prevalence rates of overweight and obesity has never been investigated. Although the Benn-index and BMI have been shown to be predictors of adult overweight [6], an index kg/m^p^ with fixed values of *p*>2 was never tested and AUCs were never presented.

Therefore, the aim of our study is to investigate 1) the relation between height and national prevalence rates of overweight and obesity in childhood between 1980, 1997, and 2009, and 2) to calculate which fixed value of *p* (2.0,2.1, …,3.0) in kg/m^p^ during childhood is most accurate in predicting adult overweight.

## Methods

### Ethics Statement

Data collection of growth studies is part of routine youth health care in The Netherlands, and is not regarded as medical research. In the Dutch nationwide surveys, oral consent was obtained from each child (and parent for children younger than 16 years) before measurement. Cooperation, or lack thereof, was registered on the questionnaire. The Medical Ethical Review Board of Leiden University Medical Centre approved of the study and the way consent was obtained. The study protocol of the Terneuzen Birth Cohort was approved by the Medical Ethics Committee of the VU University Medical Centre Amsterdam, and written informed consent was obtained from all the participants. The Harpenden study was undertaken at a time (1949 onwards) when ethical review boards did not exist.

### Materials

#### Cross-sectional data

Cross-sectional individual height and weight data were obtained from three Dutch nationwide surveys in 1980 (n = 41,805), 1997 (n = 14,500), and 2009 (n = 10,030) [18]–[22]. Mean final height was 182.0 in boys and 168.3 in girls in 1980, 184.0 in boys and 170.6 in girls in 1997, and 183.8 in boys and 170.7 in girls in 2009. Data were obtained at Well Baby Clinics, Municipal Health Services (MHS), schools and a festival (in 1997 and 2009). Children with diagnosed growth disorders and those on medication known to interfere with growth were excluded from these studies.

#### Longitudinal data

The Terneuzen Birth Cohort consists of all 2,604 children born in 1977–1986 in the city of Terneuzen in The Netherlands [23]. For 1,701 subjects, data for height and weight were obtained at Well Baby Clinics and MHS from birth until adolescence. In total, 763 subjects aged 18–28 y participated in a follow-up study that included a physical examination and a questionnaire to collect socio-demographic characteristics.

For the Dutch nationwide surveys and the Terneuzen Birth Cohort, all measurements were standardised and performed by trained health care professionals. Infants' length was measured to the nearest 0.1 cm in the supine position until 2 years of age. From 2 years of age, standing height was measured to the nearest 0.1 cm. Infants up to 15 months of age were weighed naked on calibrated baby scales. Children were weighed on calibrated mechanical or electronic step scales. Weight was rounded to the nearest 0.01 kg for infants and to the nearest 0.1 kg for older children. Older children were wearing underwear only, or a correction was made for clothes (0.4–1.0 kg).

The Harpenden Growth Study assessed the growth and maturation of several hundred children living in a children's home on the outskirts of London between 1948 and 1972 [24]–[25]. Most entered at between 3 and 5 years and stayed in the home till between 15 and 18. The home is well situated in extensive grounds, the food is excellent, and the children attended the schools in the town in the ordinary way. Measurements were made every 6 months during childhood and every three months during puberty and all measurements were made by the same observer throughout the study (RH Whitehouse). We selected the children whose measurements were made both in childhood and adulthood (n = 256).

### Data handling

SD values for height were obtained from Dutch reference charts [18], [19], [21]. Overweight and obesity were calculated according to cut-offs for BMI; 25 and 30 kg/m^2^ respectively in adults and the IOTF cut-offs in childhood [3]. Overweight prevalence rates include obesity.

### Statistical analyses

#### Cross-sectional data

To investigate the relation between height and national prevalence rates of overweight and obesity in childhood between 1980, 1997, and 2009, we used the cross-sectional data. We calculated age-sex-specific prevalence rates of overweight and obesity for children with short stature (<−1 SD), average stature ([−1,1] SD), and tall stature (>1 SD) in the three surveys. For all age years, we calculated the RR for overweight in tall versus average stature and short girls or boys in the (unweighted) pooled cross-sectional data. Then we combined adjacent age years in which the RR were all either statistically significant or not. We also investigated the differences in RR between age years to confirm our choice of age groups.

We expressed the trend in overweight between 1980 and 1997 in short and tall children by the prevalence ratio, which is the prevalence of overweight in 1997 divided by the prevalence in 1980. Similar analyses were performed for the trend between 1997 and 2009. A prevalence ratio greater than one indicates a positive trend. A log-binomial model was used with overweight as the dependent variable and survey year (1997 versus 1980 or 2009 versus 1997), height (tall versus short or average stature), and their interaction as independent variables. The interaction term models the difference in trend of overweight in tall children versus average stature or short children.

#### Longitudinal data

In order to calculate which fixed value of *p* (2.0,2.1, …,3.0) in kg/m^p^ is most accurate in predicting adult overweight, we used the Terneuzen Birth Cohort and the Harpenden Growth Study.

As a measure of diagnostic accuracy, we used the AUC. The Receiver Operation Characteristic (ROC) curve plots the sensitivity (i.e. proportion of adults with overweight who were overweight in childhood) against the 1-specificity (i.e. proportion of adults with a healthy weight who had a healthy weight in childhood). The AUC is a way to reduce ROC performance to a single value representing expected performance. An AUC varies between 0.5 and 1.0 with higher values indicating a better predictive model. The analyses were based on two-way tables with yes/no adult overweight against yes/no childhood overweight according to cut-offs for kg/m^p^. Therefore, cut-offs for childhood overweight are needed. IOTF cut-offs are available for childhood overweight when *p* = 2.0, but no cut-offs are available for *p>*2. Instead, for all *p* (2.0,2.1, …,3.0) we used cut-offs on the predicted probabilities of a logistic regression model with yes/no adult overweight as the dependent variable and the independent variables: kg/m^p^, sex, and the interaction sex with kg/m^p^ during childhood (2–13 y) per year of age.

All statistical analyses were performed using SPSS Version 20.0 for Windows (SPSS Inc., Chicago, IL, USA). P-values <0.05 (two-sided) were considered as statistically significant.

## Results

Table 1 shows the general characteristics of the cross-sectional and the longitudinal data. All studies provide large samples of children, adolescents, and adults.

**Table 1:** General characteristics of the data available for analyses.

	Cross-sectional data			Longitudinal data	
	Third Dutch Growth Study	Fourth Dutch Growth Study	Fifth Dutch Growth Study	Terneuzen Birth Cohort	Harpenden Growth Study
Number of children	41,805	14,500	10,030	763	256
Number of measurements	41,805	14,500	10,030	8,465	5,582
Age range in years	0–20	0–20	0–25	0–28	1–35
Girls (%)	48	48	52	62	47

### Cross-sectional data

The RR for overweight in tall versus short girls were not statistically significant in 2–4.9 y, 14–17.9 y olds, and significant in 5–13.9 y olds. The RR in boys were not statistically significant in 2–4.9, 15–17.9 y olds, and significant in 5–14.9 y olds. Also, the data revealed large differences in the RR between 4 and 5 y olds, and between 13 and 14 y old girls and 14 and 15 y old boys. We, therefore, obtained three age groups per sex; 2.0–4.9 y, 5.0–13.9 y and 14.0–17.9 y olds in girls and 2.0–4.9 y, 5.0–14.9 y and 15.0–17.9 y olds in boys. Table 2 shows the prevalence rates of overweight and obesity in tall, average stature, and short children and Table 3 presents their RR. Overall, tall children in the youngest and oldest age groups had no large differences in overweight and obesity rates compared to the average stature or short children. In contrast, in 5.0–13.9 y old tall girls and in 5.0–14.9 y old boys the prevalence of overweight and obesity was much higher than their shorter peers. In this age group, the strength of associations seems stronger among boys than among girls. The differences between the tall and average stature group were less pronounced.

**Table 2:** The prevalence of overweight in short (<−1 SD), average stature, and tall (>1 SD) girls and boys in the three age groups and periods.

Age (y)	Year of Growth Study	Sample size	% overweight within short children (95%CI)	% overweight within average stature children (95%CI)	% overweight within tall children (95%CI)
Girls					
2.0–4.9	1980	3,569	8.7 (6.1–12.2)	7.5 (6.5–8.6)	10.2 (8.2–12.6)
	1997	843	7.0 (3.5–13.3)	8.2 (6.1–10.8)	13.7 (8.6–20.8)
	2009	839	8.7 (4.6–15.3)	12.4 (9.9–15.4)	18.1 (12.3–25.8)
5.0–13.9	1980	7,627	3.3 (2.5–4.5)	6.9 (6.3–7.7)	10.5 (8.9–12.4)
	1997	2361	5.8 (3.8–8.8)	12.1 (10.6–13.8)	19.7 (16.0–24.0)
	2009	2,325	6.9 (4.6–10.2)	14.7 (13.1–16.6)	26.5 (22.1–31.3)
14.0–17.9	1980	3,080	9.0 (6.6–12.2)	6.7 (5.7–7.9)	6.7 (4.6–9.6)
	1997	1,215	10.5 (6.5–16.3)	8.2 (6.5–10.3)	9.1 (5.5–14.7)
	2009	926	12.2 (7.4–19.4)	11.5 (9.2–14.3)	18.2 (12.4–25.7)
Boys					
2.0–4.9	1980	3,776	6.0 (4.2–8.4)	5.4 (4.5–6.3)	4.5 (3.2–6.4)
	1997	828	4.0 (1.5–9.6)	6.6 (4.7–9.0)	12.1 (7.4–19.0)
	2009	788	5.6 (2.5–11.7)	6.0 (4.2–8.5)	13.3 (8.3–20.5)
5.0–14.9	1980	8,618	1.9 (1.3–2.7)	3.9 (3.4–4.4)	8.0 (6.5–9.8)
	1997	2,724	3.2 (1.8–5.4)	8.2 (7.0–9.6)	13.6 (10.6–17.2)
	2009	2,307	6.0 (4.0–9.0)	12.8 (11.2–14.6)	24.4 (20.2–29.2)
15.0–17.9	1980	2,512	3.9 (2.4–6.2)	5.0 (4.0–6.1)	5.0 (3.0–8.2)
	1997	1,110	7.4 (4.2–12.6)	6.7 (5.1–8.8)	8.6 (5.2–13.8)
	2009	613	6.7 (2.8–14.7)	13.4% (10.4–17.1)	12.1 (6.5–21.0)

**Table 3:** The relative risks for overweight and obesity in tall (>1 SD) versus average stature, and tall versus short stature (<−1 SD) in girls and boys in the three pooled cross-sectional data.

	Overweight	Overweight	Obesity	Obesity
	RR (95%CI)	RR (95%CI)	RR (95%CI)	RR (95%CI)
Age (y)	Tall versus average stature	Tall versus short	Tall versus average stature	Tall versus short
Girls				
2.0–4.9	1.4 (1.1–1.7)	1.4 (1.0–1.9)	2.2 (1.2–4.0)	1.2 (0.5–2.6)
5.0–13.9	1.6 (1.4–1.8)	3.5 (2.8–4.4)	1.8 (1.3–2.5)	3.9 (2.1–7.4)
14.0–17.9	1.2 (0.9–1.5)	1.0 (0.7–1.3)	0.7 (0.2–2.0)	0.8 (0.2–2.9)
Boys				
2.0–4.9	1.2 (0.9–1.6)	1.2 (0.8–1.7)	2.8 (1.4–5.7)	2.8 (0.9–8.6)
5.0–14.9	2.0 (1.7–2.3)	4.4 (3.4–5.7)	2.8 (1.9–4.2)	5.3 (2.6–11.0)
15.0–17.9	1.1 (0.8–1.5)	1.4 (0.9–2.2)	0.8 (0.3–2.3)	1.2 (0.3–4.9)

Table 4 shows the trend in overweight between 1980–1997 and 1997–2009 in short, average stature, and tall children expressed as a prevalence ratio. Regression analyses revealed that tall boys aged 2.0–4.9 y had a significantly higher positive trend compared to short boys between 1980 and 1997 (RR = 4.0,95%CI:1.38–11.9). For other age groups and in girls, no significant differences in prevalence ratios were found.

**Table 4:** The trend in overweight between 1980–1997 and 1980–2009 in short (<−1 SD), average stature, and tall (>1 SD) girls and boys expressed as a prevalence ratio.

Age (y)	Short		Average stature		Tall	
	1980–1997 Prevalence ratio (95%CI)	1997–2009 Prevalence ratio (95%CI)	1980–1997 Prevalence ratio (95%CI)	1997–2009 Prevalence ratio (95%CI)	1980–1997 Prevalence ratio (95%CI)	1997–2009 Prevalence ratio (95%CI)
Girls						
2.0–4.9	0.8 (0.4–1.7)	1.3 (0.5–3.1)	1.1 (0.8–1.5)	1.6 (1.1–2.3)	1.4 (0.8–2.4)	1.4 (0.7–2.7)
5.0–13.9	1.8 (1.1–3.0)	1.2 (0.7–2.2)	1.8 (1.5–2.2)	1.3 (1.0–1.5)	2.1 (1.5–2.8)	1.5 (1.0–2.1)
14.0.17.9	1.2 (0.7–2.1)	1.2 (0.6–2.4)	1.2 (0.9–1.7)	1.5 (1.0–2.1)	1.4 (0.7–2.7)	2.2 (1.1–4.3)
Boys						
2.0–4.9	0.7 (0.2–1.7)	1.4 (0.4–4.7)	1.2 (0.9–1.8)	0.9 (0.6–1.5)	2.9 (1.6–5.4)	1.1 (0.5–2.3)
5.0–13.9	1.7 (0.9–3.3)	1.9 (1.0–3.8)	2.2 (1.8–2.7)	1.6 (1.3–2.0)	1.8 (1.3–2.6)	2.1 (1.4–2.9)
14.0.17.9	2.0 (1.0–4.2)	0.9 (0.3–2.5)	1.4 (1.0–2.0)	2.2 (1.5–3.2)	1.8 (0.9–3.6)	1.5 (0.6–3.3)

Positive trend when prevalence ratio >1.

Prevalence ratio =  prevalence of overweight divided by the prevalence in the previous period.

### Longitudinal data

**Figure 1:**
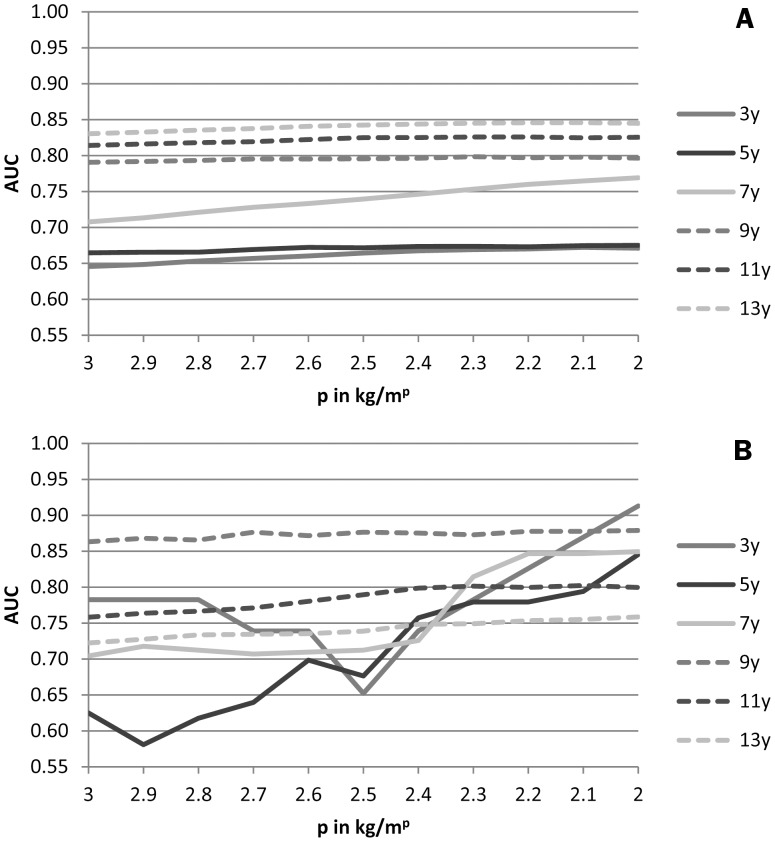
Area Under the Curves (AUC) for each *p* (2.0,2.1, …,3.0) in kg/m^p^ in childhood to predict adult overweight in the Terneuzen Birth Cohort (A) and the Harpenden Growth Study (B).

There were no differences in sensitivities and specificities between the prognostic test that used the predicted probabilities for the model that includes BMI (i.e. *p* = 2) and the IOTF cut-offs. Therefore, the predicted probabilities can be used as an alternative for cut-offs on kg/m^p^. Figure 1 shows that the AUC to predict adult overweight (slightly) increases by increasing values of *p* from 2.0 to 3.0. The ROC curves for the Harpenden Growth Study are less smooth, because the number of adults with overweight was smaller than in the Terneuzen Cohort Study. In summary, both studies show that an index *p* of 2.0 during childhood is most accurate in predicting adult overweight.

## Discussion

Three large surveys in The Netherlands show that the national prevalence rates of overweight and obesity were respectively 4 to 5 times higher in tall than in short girls aged 5.0–13.9 y and boys aged 5.0–14.9 y. Tall boys aged 2.0–4.9 y had a significantly higher positive trend compared to short boys between 1980 and 1997, but no significant trends were found in the other age groups and in girls. Our longitudinal data revealed that BMI, instead of *p*>2 in kg/m^p^, during childhood is most accurate in predicting adult overweight.

Several studies give explanations for the higher overweight prevalence in tall children. One explanation is that skeletal maturation (ultrasound bone age/chronological age) may play a role on the association between height and adiposity [16], [26]–[27]. It is shown that in pre-pubertal children the association between height and adiposity becomes weaker when adjusting by skeletal maturation, especially in girls [26]. Furthermore, increased BMI in children on a path to becoming overweight adults precedes an advancement in skeletal development and subsequently tall stature during puberty [27]. In our study, the group with tall children aged 5 to 14/15 years also includes all of the early developers who, as we know, tend to exhibit high BMI just before their adolescent growth spurt. They may influence the outcome unless we control for rate of development. To investigate this, we applied a log-binomial model with yes/no overweight as the dependent variable and tall versus short as independent variable controlled for pubic hair development SDS in 9–14 y old children in the Third and Fourth Dutch Growth Studies [28]. The results show that the risk for overweight in tall versus short children was slightly reduced after controlling for development (from RR = 2.7,95%CI:1.9–3.7 to RR = 2.3,95%CI:1.6–3.3), but it remained significant. Similar results were found for other pubertal stages in boys and girls.

Another explanation is that genes and nutrition are key determinants for both height and fatness. A large international twin study showed that genetics appear to play an increasingly important role in explaining the variation in height and BMI from early childhood to late adolescence, particularly in boys [29]. Moreover, dietary patterns that are high in energy-dense, high-fat and low-fibre foods predispose young people to later overweight and obesity [30]. Some evidence is found that genes and nutrition can influence height and fatness simultaneously [12], [31], but further research is needed. Buchan *et al.* showed that between 1988 and 2003, the positive trend in fatness seen in 3 year old children was much greater in taller children [9]. They conclude that tall stature has, therefore, become important for childhood obesity. Our study also showed a higher positive trend in overweight between 1980 and 1997 in 2.0–4.9 y old tall boys compared to short boys. However, we did not find significant differences in trends between tall, average stature, and short boys in the other age groups from 5 y onwards and in girls. The latter may also be partly explained by the sexual dimorphism regarding body composition with boys favouring greater lean mass accumulation, and females more fat mass [32]. Possibly, also an association between height and lean mass plays a role, which is congruent with the finding that the only significant prevalence ratios of overweight between 1980–1997 clusters among the boys (the very young ones), but not the girls.

Freedman *et al.* showed that height was not only associated with BMI in 5–18 year old children, but also with skinfold thickness and body fat percentage [6]. In a related study, the authors conclude that taller children are more likely to be obese in adulthood [5]. In addition, Eriksson *et al.* found that an increase in height between 2 and 11 years was also associated with impaired glucose tolerance and type 2 diabetes in adult life [10]. In a longitudinal study, Metcalf *et a*l. showed that between 7 and 12 years of age BMI and fat mass index are positively correlated with height, leptin and insulin resistance [14]. Based on these studies, one may argue that the use of BMI to approximate body fat is appropriate for both tall and short children because height and adiposity and related cardiometabolic risk are positively correlated before the age of 12 years [6], [7].

Telford and Cunningham showed that both BMI and body fat percentage are related to height in 8-year-old children and conclude that both measures may misrepresent childhood adiposity, especially in tall or short children [33]. They propose that improved diagnostic accuracy of body composition is provided by body mass/height^3^ and fat mass/body mass^1.5^. Also other measures are suggested to correct for height during childhood, such as the Benn index (kg/m^p^) with *p* varying with age and sex or the Ponderal index (kg/m^3^). Our two longitudinal data showed that from all fixed values of *p* (2.0,2.1, …,3.0) in kg/m^p^, the value of *p* = 2.0, resulting in the BMI, was most accurate in predicting adult overweight. This is in agreement with the Bogalusa heart study that reported that childhood BMI showed the strongest associations with adult BMI [6]. These results indicate that childhood BMI is an appropriate measure of predicting adult overweight and is, therefore, important to monitor.

Although there are several explanations for the higher overweight prevalence in tall children, using BMI for overweight can complicate a fair national and international comparison as height differs between populations. In The Netherlands, we observe a strong peak in overweight prevalence in pre-pubertal primary school children [22]. In 2009, the prevalence of overweight in Dutch children was 10% in 2–4 y olds, increased to 16% in 5–10 y olds, and decreased to 13% in 11–18 y olds. As the Dutch may be the tallest children in the world, part of this peak may be caused by the height difference between countries and applying international cut-offs for BMI. Furthermore, comparisons may be biased when observing nationals trends. For example, mean height of Dutch 6 year old boys has increased from 118.8 to 119.9 cm ( = +0.22 SD) between 1980 and 2009. The overweight prevalence in boys at this particular age has increased from 4% to 14%. Part of this difference in overweight prevalence may be explained by the increase in mean height. By stratifying the 1980 and 2009 prevalence rates by height, it will become clear to which extent the increase in prevalence may be explained by the increase in height in 6 year old boys.

A particular strength of our study is the consistent methodology and inclusion/exclusion criteria, and objective measurements of height and weight in the large national growth studies. A limitation of our study is that we did not investigate age groups according to biology (infancy, childhood, and adolescence) or social circumstances associations (pre-school, primary, secondary/high school children), which may reveal other relationships and trends. Another limitation of our study is that we did not study the association between height in childhood and other markers of obesity in adult life, such as waist circumference as a proxy of visceral fat. Further research is recommended to investigate this.

## Conclusions

Tall children aged 5 to 14/15 y have much higher prevalence rates of overweight and obesity than their shorter peers. In this age group, tall children did not have a significantly different trend in overweight between 1980, 1997, and 2009 compared to children who are short or have an average stature. We suggest taking into account the impact of height when evaluating trends and variations of BMI distributions between and within populations of children. Since childhood BMI is an accurate measure of predicting overweight in adulthood, monitoring childhood BMI continues to be important.
